# Characterisation of smoking behaviour across the life course and its impact on decline in lung function and all-cause mortality: evidence from a British birth cohort

**DOI:** 10.1136/jech.2007.068312

**Published:** 2008-05-01

**Authors:** S Clennell, D Kuh, J M Guralnik, K V Patel, G D Mishra

**Affiliations:** 1 MRC National Survey of Health and Development, University College and Royal Free Medical School, London, UK; 2 Laboratory of Epidemiology, Demography and Biometry, National Institute on Aging, Bethesda, Maryland, USA

## Abstract

**Objectives:**

To describe smoking trajectories from early adolescence into mid-life and to examine the effects of these trajectories on health and all-cause mortality.

**Methods:**

A nationally representative birth cohort study including 3387 men and women followed up since their birth in 1946 in England, Scotland and Wales. The main outcome measure is all-cause mortality by age 60 years and rate of decline in forced expiratory volume in 1 second (FEV_1_).

**Results:**

Eighteen per cent of the sample were categorised as lifelong smokers (smokers at all six waves at ages 20, 25, 31, 36, 43, 53 years), of whom 90% had begun smoking by age 18 years. By age 60 years, 10% of all lifelong smokers had died. They had a threefold increase in mortality rate compared with never smokers (hazard ratio (HR) 3.2, 95% confidence interval (CI) 2.1 to 4.8). For predominantly smokers (smokers for at least four of the six data collections), mortality rate remained higher than never smokers (HR 1.6, 95% CI 1.0 to 2.5). Predominantly non-smokers did not differ from those who never smoked (HR 1.3, 95% CI 0.9 to 2.0). Using the most recent smoking status available, current smokers had more than double the risk of mortality compared with never smokers (HR 2.4, 95% CI 1.6 to 3.5). Lifelong smokers and predominantly smokers had a greater rate of decline in lung function than never smokers (regression coefficients −18 ml/year, 95% CI −22 to −13; −6, 95% CI −10.3 to −1.7 respectively). For current smokers, the decline was 8.4 ml/year (95% CI −12.0 to −5.0) faster than never smokers.

**Conclusions:**

The strength and differentiation of adverse effects identified by using simplified smoking behaviours has highlighted the advantages of obtaining further information on lifelong smoking behaviour from former smokers, rather than just current smoking status.

Smoking is a major cause of global mortality and the single most important risk factor in developed countries. Total tobacco-attributable deaths are projected to rise from 5.4 million in 2005 to 6.4 million in 2013 and 8.3 million by 2030 under the baseline scenario of Mathers and Loncor.^1^ In the UK, it is estimated that each year 114 000 die from smoking-attributable causes, or about a fifth of all UK deaths.^2^ In tackling this issue, it is important to consider the relative performance of different measures for smoking behaviour over the lifespan in terms of their assessment of the strength of such adverse effects.

As the evidence for harmful effects of tobacco use has mounted, epidemiological research has progressed from simply identifying health risks associated with smoking to more detailed studies of the long-term effects of different smoking behaviours on mortality and morbidity. Previous studies that used a single baseline assessment of smoking history found risks from smoking that include: lung cancer risk,^3^ excess mortality,^4^ cardiovascular disease risk,^5^ ^6^ and poor lung function.^3^ ^7^ ^8^ Several well-known longitudinal studies have also identified the role of smoking in the development of chronic disease, in the risk of premature mortality^3^ ^9^^–^12 and in the health benefits associated with smoking cessation.^13^ Data from the American Cancer Society’s Cancer Prevention Study II (ACS CPS-II) revealed that those who quit smoking before age 50 years halved the risk of dying in the following 15 years compared with continuing smokers.^13^ Doll and colleagues reported that, among male doctors, prolonged cigarette smoking from early adult life tripled age-specific mortality rates, but cessation at age 50 years halved the hazard, and cessation at age 30 years avoided almost all of it.^10^

Accurate and detailed classification of smoking status is essential to determine the differential effects of cessation and to assist with the comparison of results across studies. There is, however, substantial variation in the assessment of smoking status and the way data are analysed. In some longitudinal studies, smoking behaviour is measured as current, or most recently available, smoking status.^14^ ^15^ This may be quantified further via retrospective data on the number of cigarettes smoked per day (intensity)^16^ and duration of smoking,^17^ with smoking behaviour calculated as their product and reported in terms of pack–years.^18^^–^21

There have also been several attempts to categorise adolescent smoking behaviour.^22^^–^24 In 2004, Audrain-McGovern and colleagues identified four adolescent smoking trajectories (never smokers, experimenters, earlier/faster adopters and later/slower smoking adopters).^25^ These smoking trajectories had significantly different rates and intensity of smoking progression,^25^ but few studies have prospective longitudinal data to follow such trajectories into adult life.

The Medical Research Council National Survey of Health and Development (NSHD) is a population-based birth cohort study that provides an opportunity to study lifelong smoking behaviour and its influence on mid-life health outcomes.^26^ The aims of this paper are: (1) to describe the main smoking trajectories in this cohort from early adolescence into mid-life; (2) to examine the relationships between these smoking trajectories and adult all-cause mortality to age 60 years and rate of change in lung function from age 43 to 53 years; and (3) to compare the strength of these relationships with those found using the most recent smoking status available and with those from smoking status assessed at 25 years of age.

## METHODS

The NSHD is a social class stratified birth cohort of 5362 births (2815 men and 2547 women) in the first week of March 1946 in England, Scotland and Wales. There have been 21 follow-ups of the main cohort since their birth up to the most recent contact in 1999 at age 53 years. A total of 3035 cohort members (1472 men, 1563 women) provided information at age 53 years. This corresponds to a participation rate of 70.5% among those still alive and resident in England, Wales or Scotland, and 89.6% for whom contact was attempted. Contact was not attempted for individuals who had previously refused to take part (n = 648), were untraced since last contact at 43 years (n = 266), were living abroad (n = 583) or had already died (n = 476). Those who responded at age 53 years were in most respects representative of the national population of a similar age.^27^ Ethics approval for this study was given by the North Thames Multicentre Research Committee.

**Figure 1:**
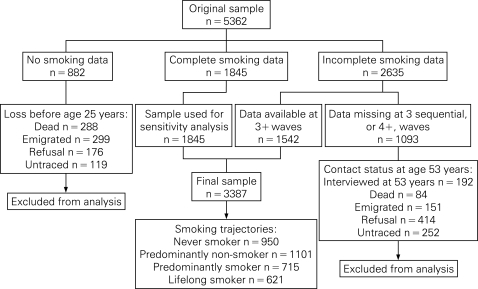
Smoking data follow-up in the Medical Research Council National Survey of Health and Development.

Current cigarette smoking status (“yes”, “no”) and the number of cigarettes smoked per day were obtained in six waves (at ages 20, 25, 31, 36, 43, 53 years). Cohort members who provided an affirmative response to being current cigarette smokers, regardless of the quantity of cigarettes smoked per day, were classified as “smokers”, while those who provided a negative response were classified as “non-smoker”. From age 36 onwards, research nurses obtained information on smoking during a home interview, while data from earlier years were collected by postal questionnaire. A total of 1845 (34%) participants provided information on smoking at all six waves, 2635 (49%) had missing information for at least one wave, and 882 (17%) failed to provide any smoking data (fig 1).

The sample for this analysis comprised those who provided data for at least three waves (n = 3387) and for whom missing data are not sequential (fig 1). Cohort members were classified into one of four smoking trajectories:

1. “Never smoker”: a non-smoker at all available data collections
2. “Predominantly non-smoker”: a non-smoker for at least three data collections
3. “Predominantly smoker”: a smoker at four or more of the data collections
4. “Lifelong smoker”: a smoker at all available data collections.

In cases where data were missing from one or more waves, classification was based on the smoking status at the majority of data collections. Thus, if at three out of four data collections the cohort member reported being a smoker, then they were classified as “predominantly smoker”. To permit a comparison with results from the smoking trajectories, two single time point measures were also used: the most recent smoking status available from cohort members (including from those who had died) and smoking status at age 25 years.

Smoking dosage to age 53 years was measured by the average number of cigarettes smoked per day: determined from the pack–years of smoking calculated at each data collection. Age at which the cohort member began smoking was recorded retrospectively at age 20 years.

In order to evaluate the performance of the various measures of smoking behaviour, smoking-related health outcomes, namely all-cause mortality,^3^ ^9^^–^12 and changes in lung function,^28^ ^29^ were chosen. Since age 26 years, cohort members have been flagged for death notification on the National Health Service Central Register. In order to ensure that cohort members who died had provided sufficient information on smoking behaviour, time to all-cause mortality was taken from age 36 years onwards. Between 36 and 60 years, there were 211 deaths. Lung function was available to all cohort members and was denoted by forced expiratory volume in 1 second (FEV_1_) measured at ages 43 and 53 years using the Micromedical turbine electronic spirometer, administered by a trained nurse. The rate of lung function change per year over the two time points was used in the analysis (FEV_1_ at 53 years–FEV_1_ at 43 years/10), with a decline recorded as a negative value.

### Statistical analyses

Cox proportional hazard models were used to investigate the relations between each measure of smoking behaviour and all-cause mortality.^30^ In order to obtain the hazard ratios (HRs) for all-cause mortality, follow-up time (in months) was from the cohort’s 36th birthday until the first of death, emigration or the end of March 2006, the cohort’s 60th birthday. If death had not occurred, follow-up was treated as censored. The assumption of proportional hazards (PH) was evaluated both by the inspection of the Kaplan–Meier survival curves and by testing the null hypothesis that the PH assumption holds for each measure of smoking behaviour. These were obtained from the statistical package STATA 8.2, commands *stphplot* and *stphtest* respectively.

Linear regression was used to examine the relation between rate of FEV_1_ change from age 43 to 53 years and each measure of smoking behaviour. The regression coefficients and the 95% confidence intervals (CIs) were adjusted for FEV_1_ at baseline (43 years) and height at age 53 years.^18^ As the main aim of this paper was to compare the results obtained from the various measures of smoking behaviour, rather than to examine the predictors of all-cause mortality or lung function, only results from univariable analyses were presented. All analyses were carried out on data from at least three waves (n = 3387). A sensitivity analysis was carried out by repeating all analyses on those with complete smoking status data (n = 1845). As the findings from these analyses were broadly similar, results from the larger sample size are presented here.

## RESULTS

**Figure 2:**
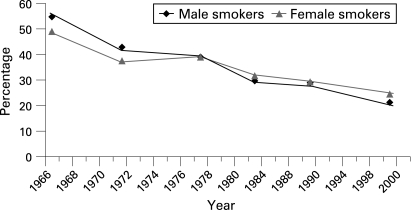
The percentage* of current smokers from age 20 years in 1966 to age 53 years in the NSHD cohort (n = 1845).

Based on the 1845 cohort members, who completed all six waves of questionnaires, the prevalence of smoking more than halved for men from 55% at age 20 years to 21% by age 53 years (fig 2). The reduction was less for women, declining from 49% at age 20 years to 24% at age 53 years. In this cohort, a higher proportion of smokers at age 25 years (p<0.001) provided incomplete data (48%), compared with the never-smoking group (31%). Those with incomplete smoking data were more likely to be male (54% incomplete vs 47% complete), have primary or lower education level (72% vs 61%) and come from a manual social class background (37% incomplete vs 29% complete). Of the 3387 cohort members who provided information for smoking trajectories, there was only one for whom mortality status was not available.

More than one in four of the cohort members (28%) were lifelong non-smokers (table 1), with almost a third (32%) of all women belonging to this group. Nearly a fifth (18%) of the sample were categorised as lifelong smokers, of whom 30% started smoking before age 16 years and 90% had begun by age 18 years. Of lifelong smokers, men were more likely to start smoking by age 16 years than women (56% vs 39%). In the predominantly non-smoking group, more than two out of three (67%) had already stopped at least once by 25 years, and only 3% were smokers at their most recent report. In addition, only one individual had smoked more than 15 cigarettes per day. In contrast, around one out of five predominantly smokers (17%) and three out of four of the lifelong smokers had 15 or more cigarettes per day. Of those in the cohort that were recorded as being former smokers for their most recent status, 30% were predominantly smokers over their lifetime.

**Table 1:** Percentage of National Survey of Health and Development cohort members in each smoking trajectory category by smoking characteristics

Smoking characteristics	Smoking trajectories n (%)			
	Never smoker (n = 950)	Predominantly non-smoker (n = 1101)	Predominantly smoker (n = 715)	Lifelong smoker (n = 621)
Age at initiation (n)				
<16 years (489)	N/A	164 (22.8)	140 (21.3)	185 (32.2)
⩾16 years (1459)	N/A	554 (77.2)	516 (78.7)	389 (67.8)
Total (1948)	N/A	718 (100)	656 (100)	574 (100)
Smoking status at 25 years (n)				
Never (920)	813 (100)	91 (9.8)	16 (2.7)	N/A
Former (742)	N/A	624 (67.2)	118 (19.6)	N/A
Current (1163)	N/A	213 (23.0)	469 (77.8)	481 (100)
Total (2825)	813 (100)	928 (100)	603 (100)	481 (100)
Most recent smoking status (n)				
Never (950)	950 (100)	N/A	N/A	N/A
Former (1531)	N/A	1065 (96.7)	466 (65.2)	N/A
Current (906)	N/A	36 (3.3)	249 (34.8)	621 (100)
Total (3387)	950 (100)	1101 (100)	715 (100)	621 (100)
Smoking dosage: average number of cigarettes per day (n)				
1–14 (1305)	N/A	557 (99.8)	587 (82.7)	161 (25.9)
15+ (584)	N/A	1 (0.2)	123 (17.3)	460 (74.1)
Total (2839)	N/A	558 (100)	710 (100)	621 (100)

N/A, not applicable.

### All-cause mortality

**Figure 3:**
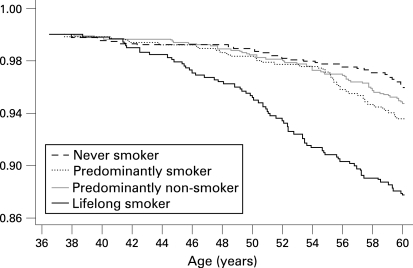
Kaplan–Meier estimates of the proportion of cohort members who are still alive by the smoking trajectories (n = 3286).

For those with smoking trajectory data, 211 study members had died between the age of 36 and 60 years: 120 men and 91 women. Of lifelong smokers, the probability of surviving past the age of 60 years was 0.88 compared with 0.96 for never smokers (fig 3). From table 2, this corresponds to a threefold increase in the hazard ratio for lifelong smokers (HR = 3.2, 95% CI 2.1 to 4.8). Predominantly non-smokers did not differ significantly from those who never smoked. For predominantly smokers, the mortality rate was almost halved compared with lifelong smokers, but remained 60% higher than never smokers.

**Table 2:** Hazard ratio and 95% confidence intervals for all-cause mortality between ages 36 and 60 years by smoking trajectories, most recent smoking status and smoking status at 25 years

	All-cause mortality HR (95% CI)		
	Men (n = 1647)	Women (n = 1639)	All (n = 3286)
Smoking trajectories (n)			
Never smoker (921)	1	1	1
Predominantly non-smoker (1086)	1.46 (0.83 to 2.56)	0.99 (0.51 to 1.92)	1.30 (0.86 to 1.99)
Predominantly smoker (692)	1.82 (1.02 to 3.25)	1.13 (0.55 to 2.35)	1.61 (1.03 to 2.50)
Lifelong smoker (587)	2.34 (1.30 to 4.19)	4.21 (2.41 to 7.33)	3.21 (2.14 to 4.79)
Most recent smoking status (n)			
Never smoker (921)	1	1	1
Former smoker (1114)	1.30 (0.74 to 2.27)	1.09 (0.56 to 2.11)	1.28 (0.84 to 1.95)
Current smoker (1251)	2.32 (1.37 to 3.92)	2.42 (1.42 to 4.13)	2.39 (1.64 to 3.48)
Smoking status at 25 years (n)			
Never smoker (917)	1	1	1
Former smoker (738)	2.35 (1.32 to 4.18)	0.56 (0.25 to 1.27)	1.50 (0.97 to 2.32)
Current smoker (1152)	1.59 (0.90 to 2.79)	2.24 (1.32 to 3.79)	1.90 (1.29 to 2.79)
Missing (479)	1.71 (0.90 to 3.25)	2.21 (1.14 to 4.26)	1.95 (1.24 to 3.07)

Using the most recent smoking status available, current smokers had more than double the risk of mortality by age 60 years compared with those who had never smoked, but less than the hazard ratio indicated by lifelong smoking status from trajectories. Former smoking status suggested a higher risk of all-cause mortality, if not statistically significant, than never smokers (HR = 1.3, 95% CI 0.8 to 1.9), which was similar to the hazard ratio indicated from trajectories by the predominantly non-smoking category. Similar hazard ratios were obtained using smoking status at age 25 years. However, male former smokers at this age had about twice the risk of dying by 60 years compared with those who had never smoked.

### Rate of change in lung function from age 43 to 53 years

Lifelong smokers had a marked decline in lung function between 43 and 53 years (−18.0 ml/per year, 95% CI −22.3 to −12.8) compared with never smokers, and three times that estimated between predominantly smokers and never smokers (table 3). No evidence for a difference in the rate of decline was found between predominantly non-smokers and never smokers. From the most recent smoking status available, the lung function of current smokers was found to have declined at a rate of 8.4 ml/year (95% CI −12.0 to −5.0) faster than never smokers. Former smokers recorded no significant difference in rate of decline in lung function relative to those who had never smoked. Similar results were obtained for rates of change in lung function using smoking status at age 25 years.

**Table 3:** Regression coefficients and 95% confidence intervals for the rate of FEV_1_ (ml/year) change between age 43 and 53 years by smoking trajectories, most recent smoking status and smoking status at 25 years*

	Regression coefficient (95% CI)†		
	Men (n = 1234)	Women (n = 1306)	All (n = 2540)‡
Smoking trajectories (n)			
Never smoker (737)	0	0	0
Predominantly non-smoker (896)	1.20 (−4.80 to 7.19)	1.30 (−3.26 to 5.76)	1.61 (−2.13 to 5.34)
Predominantly smoker (532)	−8.04 (−14.76 to −1.31)	−6.37 (−11.58 to −1.16)	−6.0 (−10.26 to −1.74)
Lifelong smoker (375)	−24.8 (−32.25 to −16.91)	−15.44 (−21.20 to −9.68)	−17.61 (−22.34 to −12.84)
Most recent smoking status (n)			
Never smoker (737)	0	0	0
Former smoker (909)	−1.56 (−7.50 to 4.38)	−2.51 (−7.15 to 2.12)	0.82 (−4.59 to 2.94)
Current smoker (894)	−12.91 (−19.16 to −6.66)	−6.13 (−10.53 to 1.73)	−8.44 (−12.21 to −4.68)
Smoking status at 25 years (n)			
Never smoker (722)	0	0	0
Former smoker (588)	1.17 (−5.61 to 7.96)	0.39 (−4.57 to 5.35)	0.5 (−3.65 to 4.71)
Current smoker (839)	−14.32 (−20.44 to −8.20)	−10.1 (−14.72 to 5.48)	−11.0 (−14.86 to −7.21)
Missing (391)	−6.34 (−13.69 to 1.02)	−23.3 (−8.20 to 3.54)	−3.40 (−8.13 to 1.33)

*Adjusted for FEV_1_ level at age 43 years (baseline) and height at age 53 years.

†Negative coefficient refers to a decline in lung function.

‡n for sample with complete data.

## DISCUSSION

Prospective data from the NSHD, spanning more than 30 years, has produced stark evidence for the differential impact of cigarette smoking behaviours on mortality rate and lung function decline. Moreover, the strength of this evidence differs according to the way smoking behaviour is characterised.

Lifelong smokers, as classified by their smoking trajectory, were at the greatest risk of all-cause mortality and, by age 60 years, more than 10% had died. Lifelong smokers also had the greatest rate of lung function decline between ages 43 and 53 years compared with those who had never smoked. Weaker effects were identified by using the single time point measures of either current (most recent) smoking status or smoking status at age 25 years. Smoking trajectories also quantified the impact of cessation by distinguishing the risk of mortality and decline in lung function between those who had been predominantly smokers throughout their life from predominantly non-smokers.

These results are broadly consistent with other studies. Doll *et al*^10^ found that smoking from early adult life among British doctors tripled age-specific mortality rates compared with lifelong non-smokers. The ACS CPS-II cohort study found that continuing smokers had around twice the overall mortality rates of those who had never smoked.^13^ There is also general agreement with other studies on the impact of quitting smoking. In the study of British doctors, it was determined that cessation by age 50 years halved the increased risk, and cessation by 30 years avoided almost all of it.^10^ Data from the ACS CPS-II also revealed that those who quit smoking before age 50 years halved the risk of dying in the following 15 years compared with continuing smokers.^13^ In the NSHD study, a gradient was found for mortality rates according to smoking duration. Predominantly non-smokers, that is those who reported being non-smokers for the majority of—rather than all—data collections, had similar all-cause mortality risk by age 60 years to lifelong non-smokers.

The marked acceleration in the rate of decline in lung function between age 43 and 53 years for lifelong smokers and predominant smokers when compared with lifelong non-smokers is consistent with other cohort studies.^28^ ^29^ However, most of these other studies have not compared the relative impact of smoking behaviour beyond that of current or former smokers.

The main limitation of this study lies in the generalisability of results due to cohort effects, as most smoking exposure had already occurred prior to the 1980s and before widespread anti-smoking programmes. Furthermore, the cohort carries risks associated with growing up at a time of heavy smoking patterns among parents.^31^ The prevalence of smoking by men and women in their thirties was slightly higher than that in the 1958 birth cohort.^32^ However, the age-related decline in smoking observed here is in broad agreement with the secular trend in the population over the last four decades.^33^^–^35 Another limitation of the study is that self-reported data do not always give an accurate assessment of smoking habits,^36^ and this may lead to misclassification and conservative estimates of the effect of smoking.^37^ Longitudinal prospective data provide the opportunity to investigate the consistency of repeated smoking behaviour reports.^38^ The main strengths of the study are the inclusion of women as well as men in a nationally representative sample, with prospective measures of smoking behaviour spanning from early adulthood to middle age.

## CONCLUSION

The methodological strategy chosen to measure smoking behaviour has implications for the strength of findings available from a study. From the NSHD, the use of smoking behaviour trajectories has identified markedly stronger adverse effects for all-cause mortality and decline in lung function for lifelong smokers than the results obtained using current smoking status or smoking status at age 25 years. Lifelong smoking behaviours also enabled the detection of differential risks for mortality and decline in lung function between predominantly smokers and predominantly non-smokers. This has highlighted the advantages of forming simplified smoking behaviour classifications by collecting detailed information from former and current smokers about their lifelong history of smoking behaviour.

**What is already known on this subject**

- The health risks as a consequence of smoking are well documented.
- Few studies have nationally representative prospective data for both men and women on smoking from early adolescence to middle age and hence are unable to assess reliably the differential effects of smoking behaviours.

**What this study adds**

- This study uses data from a nationally representative birth cohort to compare the performance of three measures of smoking status: simplified behaviours based on lifelong histories of smoking, with current smoking status and smoking status at age 25 years.
- For all-cause mortality and decline in lung function, the effects identified for lifelong smokers were stronger than those obtained for the other two smoking measures based on smoking status at a single time point.
- The use of lifelong smoking behaviours also differentiated the risks of mortality and decline in lung function between predominantly smokers and predominantly non-smokers.

**Policy implications**

In considering methodological strategies to assess the effects of smoking behaviours, this study illustrates the benefits of using information from the participant’s lifelong history of smoking.
